# Supplementary material for: The interactome of intact mitochondria by cross-linking mass spectrometry provides evidence for coexisting respiratory supercomplexes
Source: Mol Cell Proteomics. 2017 Dec 8;17(2):216–32. doi: 10.1074/mcp.RA117.000470 (PMC5795388; doi:10.1074/mcp.RA117.000470)
Supplement: Supplemental Data [file supp_RA117.000470_133922_0_supp_23978_fzffwf.zip › spectra_annotation/mito_DR_spectra_annotation/317-1-5-1-8-1.pdf]

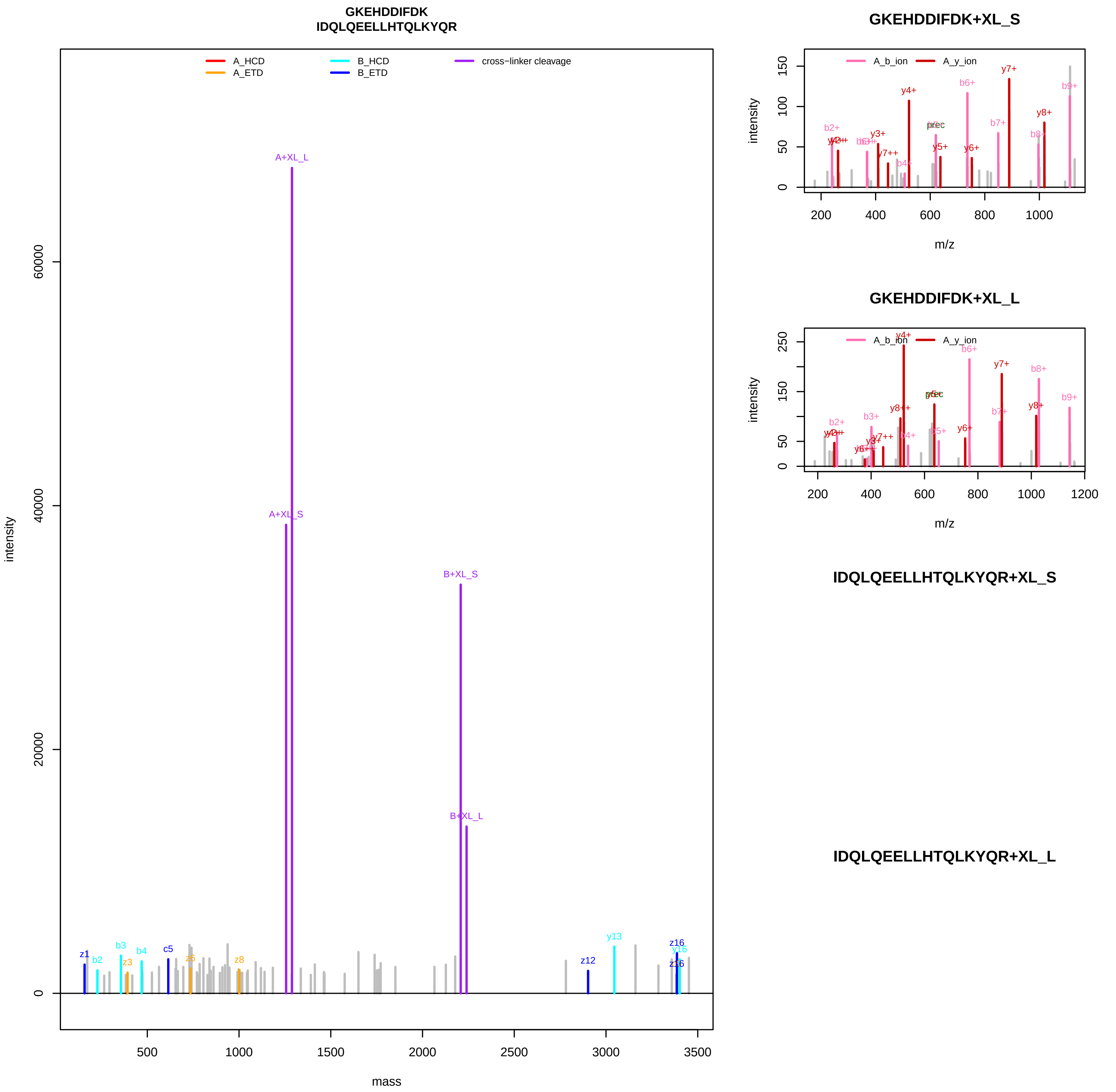
GKEHDDIFDK
IDQLQEELLHTQLKYQR
A_HCD
A_ETD
B_HCD
B_ETD
cross−linker cleavage
A+XL_L
A+XL_S
B+XL_S
B+XL_L
z1
b2
b3
z3
b4
c5
z6
z8
z12
y13
z16
y16
intensity
mass
0
20000
40000
60000
500
1000
1500
2000
2500
3000
3500
GKEHDDIFDK+XL_S
A_b_ion
A_y_ion
b2+
y3+
y4+
y5+
y6+
y7+
y7++
y8+
b4+
b6+
b7+
b8+
b9+
prec
m/z
GKEHDDIFDK+XL_L
b3+
y8++
b5+
IDQLQEELLHTQLKYQR+XL_S
IDQLQEELLHTQLKYQR+XL_L
